# Aesthetic experience as a candidate process linking group art therapy to social anxiety relief: a randomized trial with repeated process assessment

**DOI:** 10.3389/fpsyg.2026.1953607

**Published:** 2026-09-15

**Authors:** Wei Long, Yingying Luo, Jiangnan Du, Yuhuan Tang

**Affiliations:** 1 Education Center for Mental Health, Guangxi Minzu University, Nanning, Guangxi, China; 2 School of Architectural Engineering, Kaili University, Kaili, Guizhou, China; 3 Cui Zhu Campus, Nanning Tian Tao Experimental School, Nanning, Guangxi, China

**Keywords:** aesthetic experience, emotion regulation, group art therapy, longitudinal mediation, randomized controlled trial, social anxiety, university students

## Abstract

**Background:**

Direct verbal disclosure may be evaluatively demanding for university students with social anxiety. Group art therapy (GAT) offers an externally focused, materially mediated mode of engagement, but its active processes remain uncertain.

**Methods:**

In an assessor-blinded, two-arm randomized trial, 120 students with elevated social anxiety were assigned to eight weekly sessions of GAT or a structurally matched supportive discussion group (SDG). Clinical outcomes were assessed at baseline, post-intervention, and 3-month follow-up; an adapted AEQ-derived session score and two study-specific cognitive process measures were assessed after each session and analyzed exploratorily.

**Results:**

GAT produced larger LSAS-SR reductions at post-intervention (adjusted difference = −9.5, 95% CI [−15.3, −3.7], d = 0.63) and follow-up (−10.9, 95% CI [−16.7, −5.1], d = 0.71). Relative reductions calculated from group means were 35.6% and 41.4% in GAT, compared with 19.6% and 23.6% in SDG. The Condition × Session coefficient for the adapted AEQ-derived score was 0.27 (*p* < 0.001), and exploratory indirect associations were observed through self-focused attention (−0.088) and negative self-evaluation (−0.061).

**Conclusion:**

GAT was associated with greater and sustained improvement in social anxiety, while the repeated process findings support aesthetic engagement as a candidate process for further testing rather than an established causal mechanism.

## 1 Introduction

Social anxiety is common during late adolescence and emerging adulthood and can interfere with academic participation, peer relationships, and daily functioning. Recent evidence indicates a substantial burden among young people globally and among Chinese university students more broadly (Salari et al., 2024; Han et al., 2025). Because many students with elevated symptoms do not meet full diagnostic criteria or seek specialist care, accessible, low-stigma interventions that can be delivered on campus are especially valuable.

Exposure-based cognitive-behavioral therapy (CBT) remains one of the best-supported treatments for social anxiety (Hofmann and Smits, 2008; Mayo-Wilson et al., 2014). Recent group, intensive, and web-based trials have extended this evidence to varied formats and populations, including Chinese university students (Baljé et al., 2024; Wen et al., 2024; Cui and Tang, 2026). Yet these formats still ask participants to approach feared evaluation and engage in sustained verbal disclosure. Social anxiety can itself impede treatment seeking (Olfson et al., 2000), and decisions to leave therapy reflect a mixture of distress, treatment fit, perceived benefit, and practical constraints (Homan et al., 2025). The access problem is therefore not only whether an intervention is efficacious, but also whether its mode of engagement is acceptable to students whose symptoms are organized around avoidance.

Group art therapy may reduce this mismatch by organizing participation around a shared, externally focused activity rather than requiring immediate direct disclosure. Reviews and controlled trials indicate potential reductions in anxiety, although confidence in the evidence is limited by heterogeneous populations, intervention protocols, and comparison conditions (Abbing et al., 2018, 2019; Huang et al., 2025; Mizera and Krysta, 2025). Student-focused group art interventions also show promise (Yin and Ko, 2024; Li F. et al., 2025; Li J.-J. et al., 2025), but evidence specific to social anxiety and active-control comparisons remains scarce. Research on other campus delivery formats, including mobile mental health interventions, further suggests that engagement and delivery design are substantive components of intervention effectiveness (Vereschagin et al., 2024).

Cognitive models propose a social-anxiety-specific rationale for an externally focused format. Heightened self-focused attention, negatively biased self-representations, and monitoring for adverse evaluation can make direct disclosure itself feel like a social-performance task (Rapee and Heimberg, 1997; Norton and Abbott, 2016). Experimental work indicates that intensified self-focus can increase anxiety and anxious appearance, whereas directing attention toward the external interaction can reduce anxiety-related appraisals in at least some contexts (Woody, 1996; Wells and Papageorgiou, 1998; Leigh et al., 2021). Art-making may provide a concrete external target through materials, color, form, and composition, while permitting graded symbolic expression before autobiographical explanation. Evidence that externally oriented drawing can regulate affect supports plausibility (Drake and Winner, 2012), but does not establish an art-therapy mechanism. These features therefore justify testing GAT in social anxiety while keeping the proposed attentional account explicitly hypothetical.

Aesthetic experience offers a plausible candidate process for art therapy’s effects. The construct encompasses perceptual immersion, emotional arousal, personal meaning, and flow-like absorption during creative engagement (Pizzolante et al., 2024; McCrae, 2024). Artistic activity can also support emotion regulation by shifting attention, enabling symbolic processing, and altering the relationship to distressing material (Drake and Winner, 2012; Fancourt et al., 2019). Experimental evidence that art-making can reduce cortisol and perceived stress is consistent with this account (Kaimal et al., 2016). However, aesthetic experience has rarely been tested as a time-varying mediator in trials that specifically target social anxiety.

A second gap concerns temporal resolution. Most art therapy studies emphasize change from before to after treatment, which can establish whether outcomes improved but not when a candidate mechanism became active. Mechanism research requires repeated measurement and explicit tests of temporal ordering rather than inference from endpoint change alone (Kazdin, 2007). It is therefore unknown whether the therapeutic relevance of aesthetic engagement is stable across sessions or becomes stronger after group safety and familiarity with the materials have developed.

The present study addressed these gaps in a randomized trial comparing group art therapy (GAT) with a structurally matched supportive discussion group (SDG) among university students with elevated social anxiety. Clinical outcomes were assessed at baseline, post-intervention, and 3-month follow-up, while aesthetic experience and the proposed cognitive mediators were measured after each of eight sessions. This design allowed us to test treatment efficacy against an active control, examine whether growth in aesthetic experience was associated with symptom change through reductions in self-focused attention and negative self-evaluation, and determine whether the indirect effect varied across treatment stages.

## 2 Theoretical background and hypotheses

### 2.1 Evidence for group art therapy in anxiety and student populations

The evidence base for visual art therapy in anxiety has expanded, but it remains methodologically uneven. Systematic reviews report promising effects alongside substantial heterogeneity and risk-of-bias concerns (Abbing et al., 2018; Huang et al., 2025; Mizera and Krysta, 2025). Individual studies have reported reduced anxiety after art-making in students and adult clinical samples, as well as short-term physiological changes following creative activity (Sandmire et al., 2012; Kaimal et al., 2016; Abbing et al., 2019). More recent work has extended art-based approaches to international students, bereaved students, university groups, and virtual-reality sculpting (Yin and Ko, 2024; Li F. et al., 2025; Li J.-J. et al., 2025; Ding et al., 2025). Experimental work with psychedelic art illustrates the breadth of emerging creative mental health interventions, although its findings cannot be generalized to ordinary art therapy (Peng et al., 2024). Results in severe psychiatric populations are more mixed, underscoring the importance of diagnosis, dosage, and intervention process (Du et al., 2024). Collectively, this literature justifies further efficacy research but does not identify the process that distinguishes art-based treatment from supportive group contact.

### 2.2 Aesthetic experience: concept and measurement

Contemporary psychological and neuroaesthetic models treat aesthetic experience as a dynamic interaction among perceptual fluency, appraisal, emotion, embodied engagement, and meaning-making (Leder et al., 2004; Silvia, 2005; Chatterjee and Vartanian, 2014). Recent accounts further emphasize agency and the potentially transformative character of sustained aesthetic engagement (Pizzolante et al., 2024; McCrae, 2024). During art-making, absorption may redirect attention from self-monitoring to the emerging image, while sensorimotor activity offers a nonverbal route for regulating affect. Evidence that creative activities are used to regulate emotion supports this functional interpretation (Drake and Winner, 2012; Fancourt et al., 2019). The original 22-item Aesthetic Experience Questionnaire (AEQ) was developed for experiences while viewing art, used a 7-point format, and explicitly did not treat self-created art as its referent (Wanzer et al., 2020). Its six-domain structure and subsequent Polish validation (Świątek et al., 2023) do not establish validity for a 5-point post-session adaptation used during art-making and discussion-only sessions. The present score is therefore described as an adapted AEQ-derived session measure.

### 2.3 Cognitive-behavioral pathways linking aesthetic experience to social anxiety

Cognitive-behavioral models identify self-focused attention, negative self-appraisal, fear of evaluation, safety behaviors, and post-event processing as central maintenance processes in social anxiety (Rapee and Heimberg, 1997; Norton and Abbott, 2016). Daily-life evidence further links negative self-focused attention to post-event processing, while recent studies distinguish the contributions of negative and positive evaluation fears and rumination (Adamis et al., 2025; Gao et al., 2025; Hou and Shi, 2025). Aesthetic engagement could interrupt this cycle in two complementary ways. First, attention to materials, color, form, and the developing artwork may temporarily reduce inward monitoring. Second, an external artistic medium can create psychological distance from threatening material, allowing expression without immediate, explicit self-disclosure. These pathways are theoretically coherent, but direct session-level tests in socially anxious students are limited.

### 2.4 Research gaps and hypotheses

Outcome evidence alone cannot determine whether aesthetic experience is an active ingredient, a correlate of improvement, or a byproduct of successful participation. Following recommendations to study mechanisms with temporally informative designs (Kazdin, 2007), we tested four hypotheses. H1: GAT would produce greater reductions in social anxiety than SDG at post-intervention and follow-up. H2: aesthetic experience would increase more strongly across sessions in GAT than in SDG. H3: growth in aesthetic experience would be associated with reductions in social anxiety through parallel reductions in self-focused attention and negative self-evaluation. H4: the indirect effect would be strongest during the deepening stage, after initial adaptation but before the intervention shifted toward integration and narrative meaning-making.

## 3 Materials and methods

### 3.1 Study design

This study used a two-arm, parallel-group randomized controlled design with blinded outcome assessment and a nested session-level process component. Reporting was aligned with CONSORT principles for parallel-group trials (Schulz et al., 2010), and a completed CONSORT checklist has been supplied as Supplementary Material. Clinical outcomes were assessed at baseline (T0), immediately after the eight-session intervention (T9), and at 3-month follow-up (T10). The adapted AEQ-derived session score and proposed process variables were assessed after each session (T1-T8).

The design combined between-group estimation of treatment effects with within-person tracking of a proposed process. Participants were allocated to GAT or to an SDG matched on session frequency, duration, group size, facilitator configuration, and group format. The conditions differed in their primary mode of engagement: art-making and aesthetic reflection in GAT versus structured verbal discussion in SDG. The active control was intended to reduce, although not eliminate, explanations based on nonspecific group factors such as attention, cohesion, and interpersonal contact. Because SDG was itself an active intervention, the contrast estimates the comparative effect of two intervention packages rather than the isolated effect of art-making or aesthetic experience.

### 3.2 Participant recruitment and screening

Participants were recruited during the 2025 summer semester at a university in Jiangsu Province, China, through the campus mental health center, public flyers, and a psychology participant pool. Recruitment materials stated that prior visual-art training was not required. Screening used the Liebowitz Social Anxiety Scale-Self-Report (LSAS-SR), with scores of 30 or higher indicating clinically meaningful symptoms (Liebowitz, 1987; Rytwinski et al., 2009). Students above the threshold completed a study-specific, clinician-administered eligibility interview covering the prespecified inclusion and exclusion criteria. Inclusion criteria were full-time enrollment, age 18–25 years, written informed consent, and availability for eight weekly sessions and all assessments. Exclusion criteria were an acute major depressive episode, psychotic disorder, substance dependence, concurrent structured psychotherapy, prior systematic art therapy, visual impairment that precluded art-making, or an unstable psychotropic medication dosage.

### 3.3 Sample size estimation

Sample size was estimated in G*Power 3.1 (Faul et al., 2009) using effect sizes reported in prior syntheses of CBT for anxiety and social anxiety (Hofmann and Smits, 2008; Mayo-Wilson et al., 2014). With a two-sided alpha of 0.05, power of 0.80, and a repeated-measures Group × Time test, the estimated minimum was 48–50 participants per arm. To allow for attrition in this process-intensive design, the target sample was increased to 120 participants (60 per arm).

### 3.4 Randomization and blinding

Participants were assigned 1:1 using computer-generated block randomization with alternating block sizes of four and six. A research assistant who was not involved in screening, treatment delivery, or outcome assessment generated and concealed the allocation sequence in sequentially numbered, opaque, sealed envelopes. Assessors who administered the T0, T9, and T10 measures remained unaware of allocation. Participants and facilitators could not be blinded because the intervention was experiential.

Figure 1 shows participant flow. Of 158 students assessed, 38 were excluded and 120 were randomized equally to GAT and SDG (n = 60 per group). Five GAT participants and eight SDG participants did not complete the T9/T10 assessment sequence, leaving 55 and 52 participants, respectively, with complete follow-up data. All randomized participants were retained in the primary intention-to-treat (ITT) analysis.

**Figure 1:**
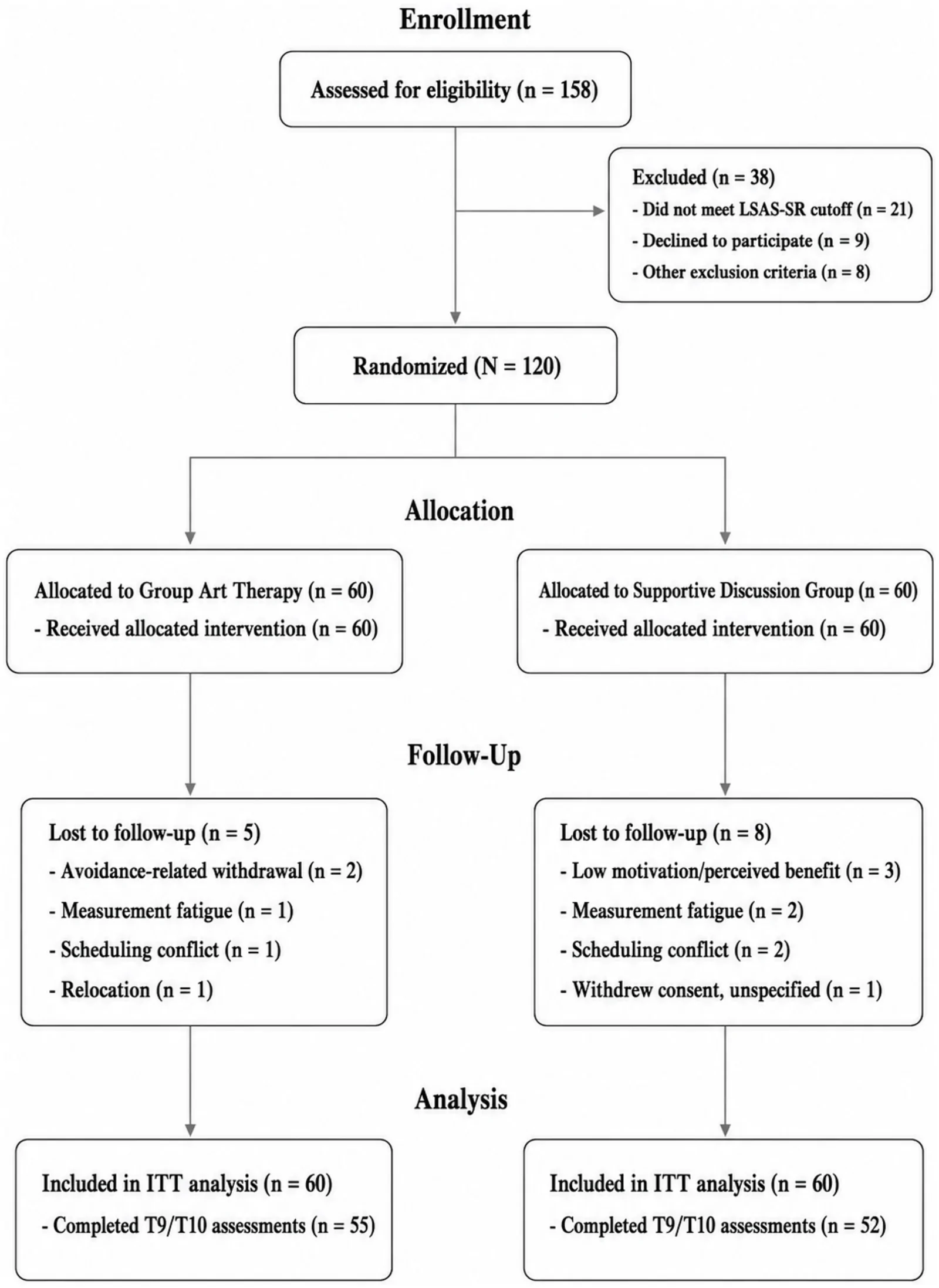
CONSORT flow diagram showing screening, allocation, follow-up, and inclusion in the intention-to-treat analysis.

### 3.5 Interventions

*Group Art Therapy (GAT)*. The intervention integrated humanistic-expressive art therapy principles with aesthetic distancing and flow concepts. Groups of six to eight participants met weekly for 90 min across eight consecutive weeks and were co-led by a credentialed art therapist and a co-facilitator. Each session followed a manualized sequence: check-in and mood rating (5 min), sensory or perceptual warm-up (10 min), thematic art-making (40 min), aesthetic viewing and sharing (25 min), and group feedback and a closing ritual (10 min). During sharing, participants were invited to describe the qualities and experience of their artwork before discussing personal anxiety, thereby preserving a degree of therapeutic distance.

The eight sessions were organized into three prespecified stages: adaptation (Sessions 1–2), which emphasized safe exploration of materials; deepening (Sessions 3–5), which used self-portrait variation and emotional color mapping to foster absorption and embodied processing; and integration (Sessions 6–8), which emphasized collaborative creation, narrative meaning-making, and social connection.

*Supportive Discussion Group (SDG)*. The SDG was selected as a structurally matched active comparator grounded in supportive group processes and CBT-informed reflection. It matched GAT on session number and duration, group format, facilitator contact, peer interaction, opportunities for emotional disclosure, and structured engagement, thereby reducing explanations based solely on time, attention, and group support (Mohr et al., 2009). SDG omitted art materials and art-making tasks. It therefore did not match sensory and material engagement, absorption in making, aesthetic appraisal, symbolic externalization, or embodied interaction with an artwork. Because structured emotion-sharing and CBT-informed prompts could themselves influence anxiety, between-condition effects estimate the incremental benefit of the multicomponent GAT program relative to SDG, not the isolated causal effect of aesthetic experience.

Treatment fidelity was monitored from video recordings using a manualized adherence checklist scored by independent raters. Sessions falling below the prespecified 85% review threshold were flagged for supervisory review, and facilitators received supervision every 2 weeks to limit treatment drift.

### 3.6 Measures

Clinical outcomes were administered at T0, T9, and T10; session-level process measures were completed immediately after T1-T8. Table 1 summarizes the instruments. Established measures included the LSAS-SR (Liebowitz, 1987; Rytwinski et al., 2009), Social Interaction Anxiety Scale (SIAS) (Mattick and Clarke, 1998), Social Avoidance and Distress Scale (SADS) (Watson and Friend, 1969), State–Trait Anxiety Inventory-State subscale (STAI-S) (Spielberger et al., 1983), Patient Health Questionnaire-9 (PHQ-9) (Kroenke et al., 2001), and the short Group Climate Questionnaire (MacKenzie, 1983). Session-specific aesthetic engagement was represented by a 22-item score adapted from the viewing-art AEQ to the study’s 5-point, immediate post-session format (Wanzer et al., 2020; Świątek et al., 2023). Because the administration context, time frame, and response format differed from the validated original instrument, we refer to it as an adapted AEQ-derived session score rather than as the original AEQ.

**Table 1:** Summary of measures and assessment schedule.

Instrument	Construct measured	Items	Response format	Assessment	Source/psychometric evidence
Liebowitz Social Anxiety Scale-Self-Report (LSAS-SR)	Social anxiety severity (fear and avoidance across social/performance situations)	24	4-point (0–3)	T0, T9, T10	Liebowitz (1987); Rytwinski et al. (2009)
Social Interaction Anxiety Scale (SIAS)	Cognitive/affective anxiety in dyadic and group interaction	20	5-point (0–4)	T0, T9, T10	Mattick and Clarke (1998)
Social Avoidance and Distress Scale (SADS)	Behavioral avoidance and subjective distress in social situations	28	True/False (dichotomous)	T0, T9, T10	Watson and Friend (1969)
State–Trait Anxiety Inventory-State subscale (STAI-S)	Transient state anxiety	20	4-point (1–4)	T0, T9, T10	Spielberger et al. (1983)
Adapted AEQ-derived session score	Session-specific aesthetic engagement (exploratory)	22	5-point (1–5)	T1-T8 (post-session)	Original viewing-art AEQ: Wanzer et al. (2020); Polish validation: Świątek et al. (2023); current 5-point post-session use not independently validated
Self-Focused Attention Scale (SFAS)	Degree of inward attentional focus during social engagement	9	5-point (1–5)	T1-T8 (post-session)	Study-specific brief state measure grounded in cognitive models of self-focused attention (Rapee and Heimberg, 1997; Norton and Abbott, 2016); pilot-tested for clarity.
State Negative Self-Evaluation Scale (brief)	Momentary negative self-appraisal following group activity	6	5-point (1–5)	T1-T8 (post-session)	Study-specific brief state measure; pilot-tested for clarity and used as an exploratory process measure.
Group Climate Questionnaire-Short Form (GCQ-S)	Perceived group engagement and climate (nonspecific process factor)	12	5-point (1–5)	T1-T8 (post-session)	MacKenzie (1983)
Patient Health Questionnaire-9 (PHQ-9)	Depressive symptom severity (covariate)	9	4-point (0–3)	T0	Kroenke et al. (2001)
Prior Art-Experience Questionnaire	Self-reported prior artistic training (covariate)	N/A	5-point (1–5)	T0	Investigator-developed; descriptive covariate

Repeated adapted AEQ-derived, self-focused-attention, and negative-self-evaluation scores constituted the process data for the longitudinal mediation analysis. The 9-item self-focused-attention and 6-item negative-self-evaluation measures were study-specific brief state instruments keyed to the just-completed session and were pilot-tested for clarity. Because these measures and the 5-point post-session AEQ adaptation were not independently validated in this context, the process analyses were treated as exploratory. Group climate was included as a time-varying nonspecific process covariate.

### 3.7 Attrition and reasons for withdrawal

Thirteen of 120 randomized participants (10.8%) did not complete the T9 assessment. Because social avoidance could plausibly influence continued participation, reasons for withdrawal were recorded descriptively and baseline predictors of attrition were examined in an exploratory analysis.

In GAT, two participants withdrew around Sessions 2–3 and reported anticipatory distress related to artwork sharing. One withdrew after Session 5 because of repeated-assessment burden, one reported a scheduling conflict, and one relocated.

In SDG, three participants reported limited perceived benefit from the discussion-only format, two reported repeated-assessment burden, two reported scheduling conflicts, and one withdrew consent without giving a reason.

These descriptions were not treated as causal explanations for attrition. Section 4.2 reports an exploratory penalized logistic regression comparing baseline characteristics of participants who did and did not complete follow-up.

### 3.8 Data analysis

Analyses were conducted in R 4.5.0 and SPSS 26.0. SPSS was used for descriptive statistics and baseline comparisons, while multilevel and structural models were fitted in R using the lme4 and lavaan packages.

Primary treatment effects (H1) were estimated with multilevel models in which repeated assessments were nested within participants. The Group × Time interaction was the main test of differential LSAS-SR change, and intention-to-treat analysis was primary. Missing outcomes were handled using multiple imputation incorporating allocation and observed baseline clinical variables (White et al., 2011). Because dropout was associated with observed baseline LSAS-SR and SADS scores, the imputation model included these baseline clinical variables. Complete-case estimates were examined as a sensitivity analysis. Secondary outcomes were interpreted as exploratory, with no formal multiplicity adjustment.

H2 and H3 were examined as exploratory process analyses using a longitudinal parallel mediation framework (Bauer et al., 2006). The model specified paths from condition to growth in the adapted AEQ-derived session score, from score growth to changes in self-focused attention and negative self-evaluation, and from those changes to LSAS-SR change. Indirect effects were evaluated using 5,000 bias-corrected bootstrap resamples. Because the proposed mediators were not experimentally manipulated and were self-reported, indirect effects were interpreted as statistical associations rather than causal mediation.

H4 was examined using a piecewise specification corresponding to the adaptation, deepening, and integration stages defined in Section 3.5. Stage-specific indirect effects were estimated and compared pairwise to evaluate whether the magnitude of the indirect association differed across treatment stages.

Baseline predictors of dropout were examined with Firth-penalized logistic regression to reduce small-sample and separation bias because only 13 participants withdrew (Heinze and Schemper, 2002). Given the small number of dropout events, this analysis was interpreted as exploratory.

Analytic approach. Primary clinical outcomes, exploratory process models, stage-specific analyses, attrition analyses, and sensitivity analyses were interpreted according to the analytic framework described above, with causal claims avoided for the observational process pathways.

### 3.9 Ethics

The protocol was reviewed and approved by the Ethics Committee of Guangxi Minzu University (approval no. 202610608001; approval effective January 1, 2025 to January 1, 2026). All participants provided written informed consent, including consent for repeated session-level assessments, and could withdraw without affecting access to usual university mental health services. A crisis-response protocol included facilitator risk assessment and referral to campus counseling. SDG participants were offered access to the GAT protocol after T10.

## 4 Results

The analyses below report the prespecified clinical and exploratory process outcomes. Clinical outcomes are interpreted according to the randomized comparison, whereas session-level process and indirect-effect analyses are treated as exploratory and are not extended beyond the prespecified analytic framework.

### 4.1 Participant flow and baseline characteristics

The randomized sample comprised 120 participants, with 60 assigned to each condition. Five GAT participants and eight SDG participants did not complete T9/T10, leaving 55 and 52 complete follow-up cases, respectively. All 120 participants contributed to the primary ITT analysis through the prespecified missing-data procedure.

Table 2 presents baseline characteristics. No statistically detectable between-group differences were observed for demographic variables, social anxiety, interaction anxiety, avoidance and distress, state anxiety, depressive symptoms, or prior art experience (all *p* > 0.19). Absolute standardized mean differences were below 0.25. Mean baseline LSAS-SR scores were 71.9 (SD = 14.5) in GAT and 69.4 (SD = 13.8) in SDG.

**Table 2:** Baseline demographic and clinical characteristics by group (*N* = 120).

Variable	GAT (*n* = 60)	SDG (*n* = 60)	Test statistic	*p*	SMD
Age, years, M (SD)	20.2 (1.8)	20.5 (1.7)	t = 0.93	0.353	−0.17
Female, n (%)	38 (63.3)	34 (56.7)	χ^2^ = 0.55	0.459	0.14
Academic year, M (SD)	2.3 (1.0)	2.5 (1.1)	t = 1.02	0.31	−0.19
LSAS-SR, M (SD)	71.9 (14.5)	69.4 (13.8)	t = 0.98	0.328	0.18
SIAS, M (SD)	53.0 (9.5)	50.8 (8.9)	t = 1.30	0.196	0.24
SADS, M (SD)	18.0 (4.3)	17.1 (4.5)	t = 1.11	0.269	0.2
STAI-S, M (SD)	49.2 (7.8)	47.3 (8.1)	t = 1.29	0.199	0.24
PHQ-9, M (SD)	9.2 (3.5)	8.5 (3.6)	t = 1.06	0.291	0.2
Prior art experience, M (SD)	1.8 (0.9)	1.9 (1.0)	t = 0.57	0.572	−0.11

SMD, standardized mean difference.

### 4.2 Exploratory analysis of attrition

Thirteen participants (10.8%) did not complete the T9-T10 assessment sequence. Given the small number of events, the exploratory analysis used Firth-penalized logistic regression rather than conventional maximum-likelihood estimation.

Participants who withdrew had higher baseline LSAS-SR scores than completers (M = 78.2, SD = 12.4 vs. M = 69.6, SD = 14.0) and higher SADS scores (M = 20.0, SD = 3.9 vs. M = 17.2, SD = 4.4). Each one-point increase in baseline LSAS-SR was associated with higher odds of dropout (OR = 1.05, 95% CI [1.01, 1.10], *p* = 0.027); the corresponding estimate for SADS was OR = 1.13 (95% CI [1.01, 1.27], *p* = 0.032).

After adjustment for baseline symptoms, condition was not statistically associated with dropout (OR = 1.59, 95% CI [0.48, 5.24], *p* = 0.445), nor was baseline PHQ-9 score (*p* = 0.687). These exploratory results suggest that missingness was associated with observed baseline severity. The primary imputation model therefore included baseline clinical variables (Table 3).

**Table 3:** Baseline characteristics and penalized logistic regression estimates for dropout.

Predictor	Dropouts (*n* = 13)	Completers (*n* = 107)	OR	95% CI	*p*
Baseline LSAS-SR	78.2 (12.4)	69.6 (14.0)	1.05	[1.01, 1.10]	0.027
Baseline SADS	20.0 (3.9)	17.2 (4.4)	1.13	[1.01, 1.27]	0.032
SDG condition, n (%)	8 (61.5)	52 (48.6)	1.59	[0.48, 5.24]	0.445
Baseline PHQ-9	9.3 (3.7)	8.8 (3.5)	1.03	[0.89, 1.19]	0.687

Dropout was coded 1 and completion 0; OR > 1 indicates higher odds of dropout.

### 4.3 Treatment outcomes

Primary treatment effects were estimated with multilevel models including all 120 randomized participants. Because the intervals between T0, T9, and T10 were unequal, Time was modeled as a categorical factor.

LSAS-SR scores decreased in both groups, with a larger reduction in GAT. In GAT, means decreased from 71.9 (SD = 14.5) at T0 to 46.3 (SD = 14.7) at T9 and 42.1 (SD = 14.8) at T10. In SDG, corresponding means were 69.4 (SD = 13.8), 55.8 (SD = 15.4), and 53.0 (SD = 15.8).

The adjusted between-group difference was −9.5 points at T9 (95% CI [−15.3, −3.7], *p* = 0.002; d = 0.63) and −10.9 points at T10 (95% CI [−16.7, −5.1], *p* < 0.001; d = 0.71). The Group × Time interaction was statistically significant (*p* < 0.001), indicating a larger and sustained reduction in GAT.

*Secondary outcomes showed the same direction of effect*. Group × Time interactions were statistically significant for SIAS (*p* = 0.003), SADS (*p* = 0.006), and STAI-S (*p* = 0.011). At T9 and T10, GAT participants reported lower interaction anxiety, avoidance and distress, and state anxiety than SDG participants. These *p* values were not adjusted for multiplicity and are interpreted as exploratory; effect sizes and confidence intervals are emphasized (Table 4).

**Table 4:** Multilevel model estimates for clinical outcomes.

Outcome/time	GAT, M (SD)	SDG, M (SD)	Adjusted difference	95% CI	Cohen’s d	*p*
LSAS-SR						Group × Time < 0.001
T0	71.9 (14.5)	69.4 (13.8)	2.5	[−2.6, 7.6]	0.18	0.328
T9	46.3 (14.7)	55.8 (15.4)	−9.5	[−15.3, −3.7]	0.63	0.002
T10	42.1 (14.8)	53.0 (15.8)	−10.9	[−16.7, −5.1]	0.71	< 0.001
SIAS						Group × Time = 0.003
T0	53.0 (9.5)	50.8 (8.9)	2.2	[−1.2, 5.6]	0.24	0.196
T9	34.8 (9.1)	40.4 (9.6)	−5.6	[−9.1, −2.1]	0.6	0.002
T10	32.7 (8.8)	38.6 (9.3)	−5.9	[−9.3, −2.5]	0.65	< 0.001
SADS						Group × Time = 0.006
T0	18.0 (4.3)	17.1 (4.5)	0.9	[−0.7, 2.5]	0.2	0.269
T9	11.2 (4.0)	13.6 (4.3)	−2.4	[−4.0, −0.8]	0.58	0.004
T10	10.1 (3.8)	12.8 (4.2)	−2.7	[−4.2, −1.2]	0.68	< 0.001
STAI-S						Group × Time = 0.011
T0	49.2 (7.8)	47.3 (8.1)	1.9	[−1.0, 4.8]	0.24	0.199
T9	37.9 (7.4)	41.9 (7.8)	−4.0	[−6.8, −1.2]	0.53	0.006
T10	36.5 (7.2)	40.7 (7.6)	−4.2	[−7.0, −1.4]	0.57	0.004

Negative adjusted differences favor GAT. Cohen’s d is reported as an absolute endpoint contrast; direction is indicated by the adjusted difference. T0 was the reference time point. Secondary-outcome p values are exploratory and unadjusted for multiplicity.

*Clinical interpretation*. Relative reductions calculated from the group means were 35.6% at T9 and 41.4% at T10 in GAT, compared with 19.6% and 23.6% in SDG. In a psychotherapy sample with diagnosed social anxiety disorder, a 28% LSAS-SR reduction best identified treatment response and a score of 35 or lower best identified remission status (von Glischinski et al., 2018).

Figure 2A shows LSAS-SR trajectories. Baseline means were similar across conditions, whereas the decline from T0 to T9 was steeper in GAT and the between-group difference persisted through T10.

**Figure 2:**
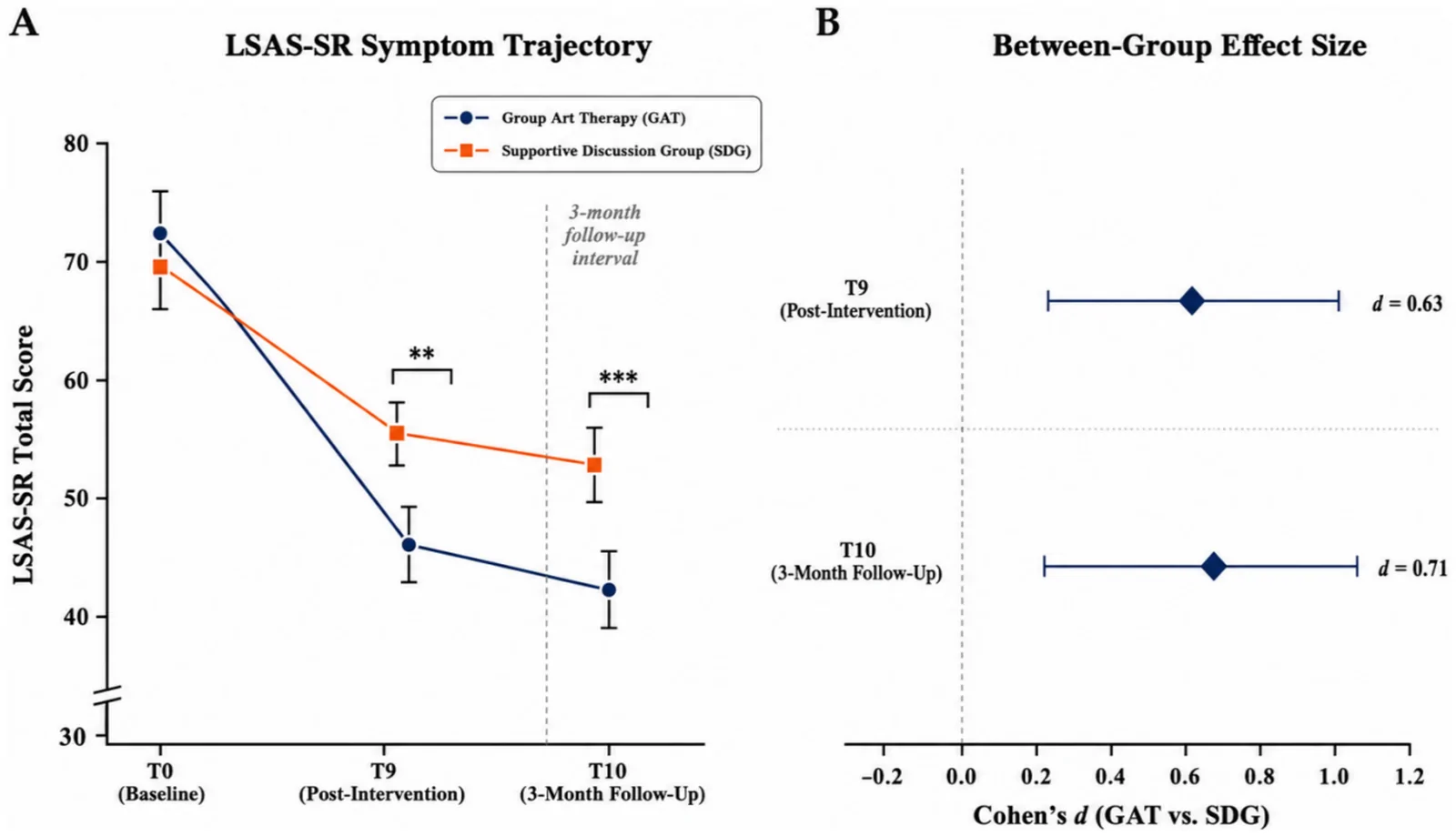
LSAS-SR outcomes. **(A)** Mean symptom trajectories from baseline to 3-month follow-up. **(B)** Standardized between-group effects at post-intervention and follow-up.

Figure 2B shows the standardized between-group effects: d = 0.63 at T9 and d = 0.71 at T10. Both estimates favored GAT.

### 4.4 Aesthetic-experience trajectories and longitudinal mediation

Figure 3 shows divergent adapted AEQ-derived session-score trajectories across the eight sessions (exact numerical session-specific means and standard deviations are provided in Supplementary Table S1). Scores increased steadily in GAT, whereas SDG scores remained comparatively stable. The between-condition divergence became most pronounced during the deepening stage and remained evident through the integration stage.

**Figure 3:**
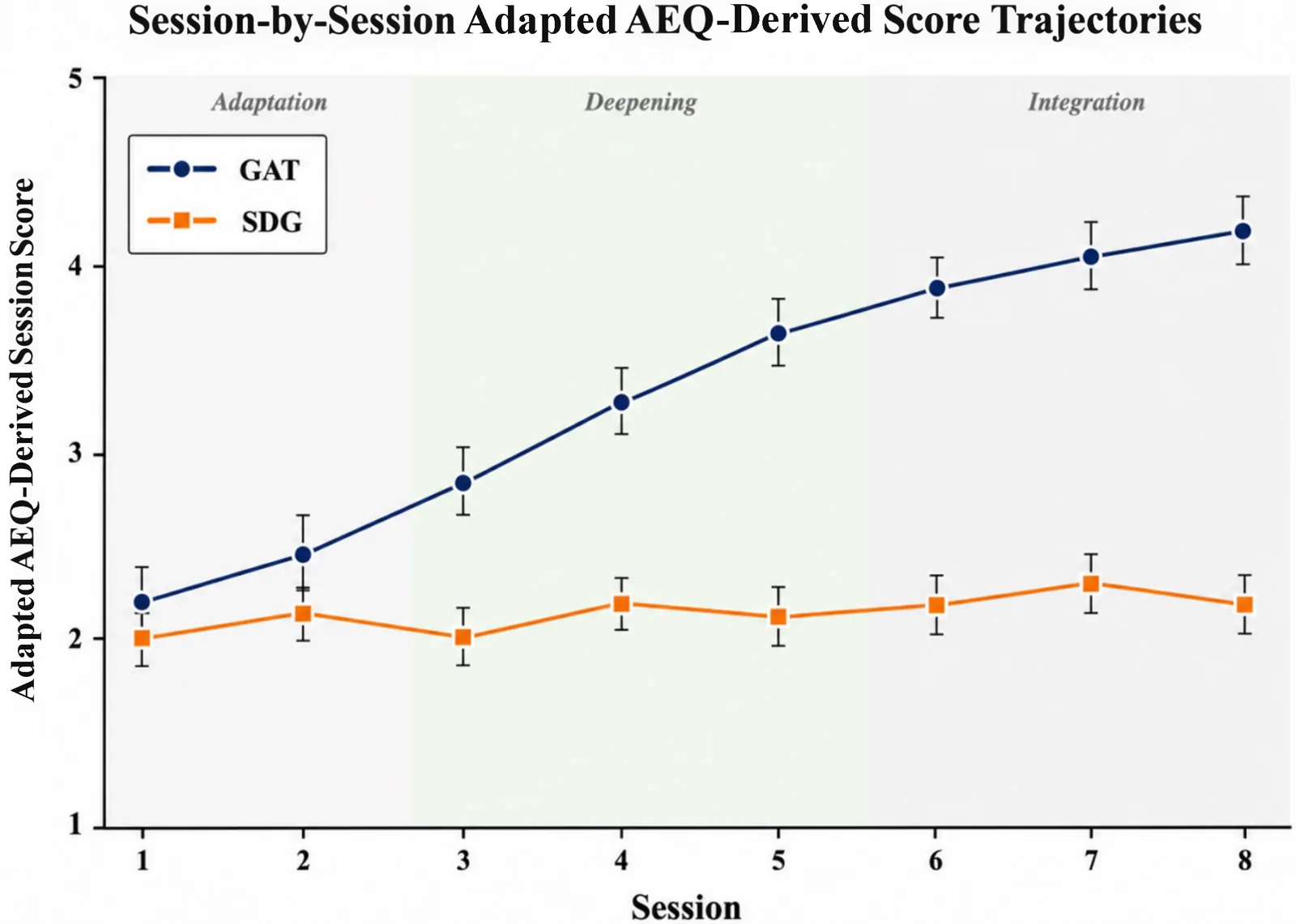
Session-by-Session Adapted AEQ-Derived Score Trajectories of the adapted AEQ-derived session score in the GAT and SDG conditions.

The Condition × Session coefficient for the adapted session score was b = 0.27 (SE = 0.03, *p* < 0.001), indicating a steeper increase across sessions in GAT than in SDG and supporting H2.

Group climate was comparatively stable. The Condition × Session coefficient for group climate was not statistically significant (b = 0.02, SE = 0.03, *p* = 0.510), and mean session-level group climate was not independently associated with LSAS-SR change in the mediation model (β = −0.047, *p* = 0.534). Adjustment for group climate changed the main AEQ-related coefficients by less than 5%.

The longitudinal parallel mediation model showed acceptable fit (χ^2^/df = 1.34, CFI = 0.968, TLI = 0.956, RMSEA = 0.046, SRMR = 0.038). Assignment to GAT was associated with greater growth in the adapted session score (β = 0.412, *p* < 0.001), which in turn was associated with reductions in self-focused attention (β = −0.331, *p* < 0.001) and negative self-evaluation (β = −0.298, *p* < 0.001).

Reductions in self-focused attention (β = 0.372, *p* < 0.001) and negative self-evaluation (β = 0.336, *p* < 0.001) were each associated with greater LSAS-SR improvement.

The indirect effect through self-focused attention was −0.088 (95% CI [−0.142, −0.041], *p* < 0.001), and the indirect effect through negative self-evaluation was −0.061 (95% CI [−0.108, −0.021], *p* = 0.003). The combined indirect effect was −0.149 (95% CI [−0.219, −0.086], *p* < 0.001).

The standardized total effect of condition on LSAS-SR change was β = −0.261 (*p* = 0.028), while the residual direct effect after inclusion of the process variables was β = −0.121 (*p* = 0.073). The combined unstandardized indirect effect was −0.149.

Figure 4 graphically summarizes the parallel process model reported in Table 5, including the associations between treatment condition, growth in the adapted AEQ-derived session score, changes in self-focused attention and negative self-evaluation, and LSAS-SR change. Assignment to GAT was associated with greater growth in the adapted AEQ-derived session score; greater score growth was associated with reductions in self-focused attention and negative self-evaluation and, in turn, greater LSAS-SR improvement.

**Figure 4:**
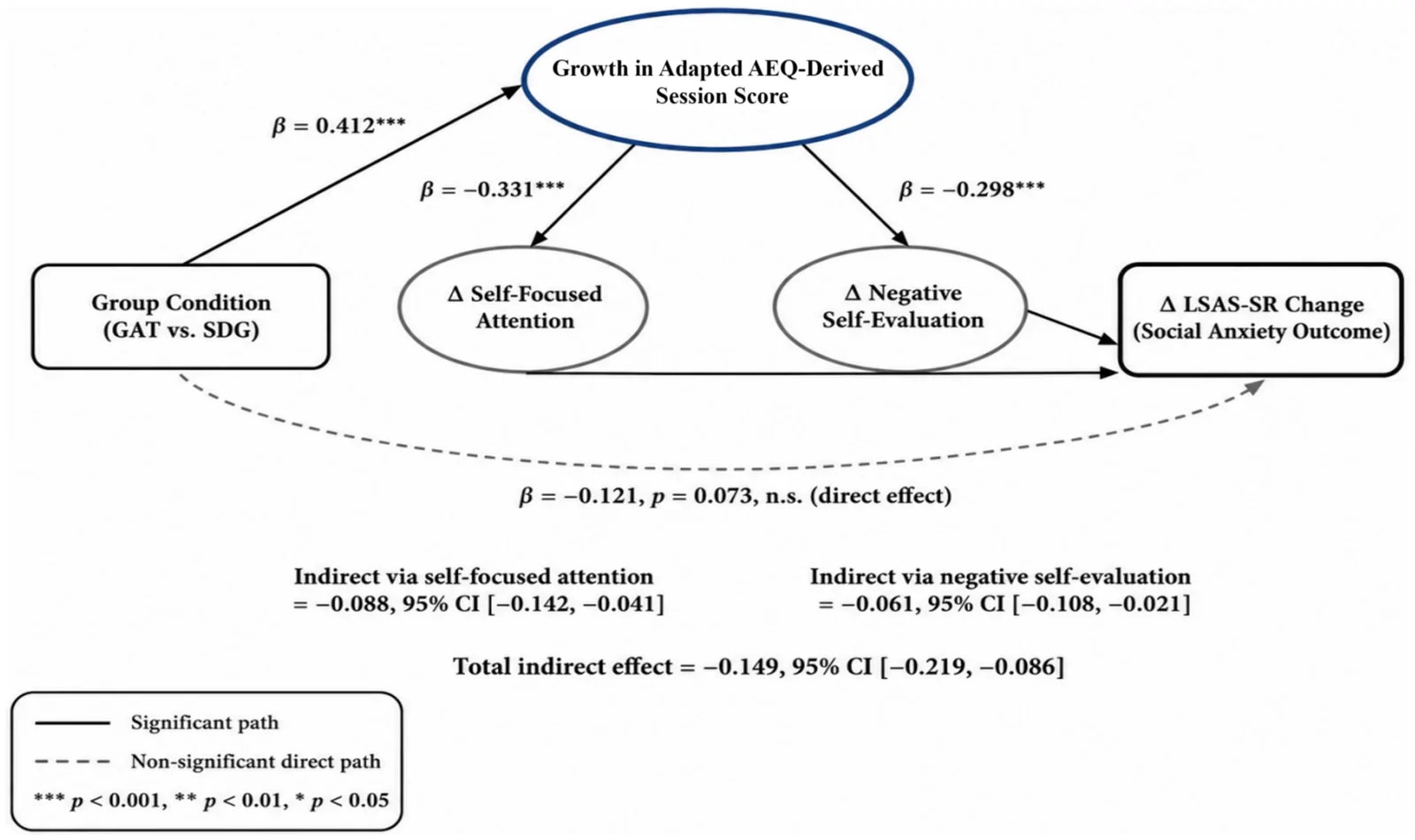
Graphical summary of the longitudinal parallel mediation model. Solid paths indicate statistically significant associations.

**Table 5:** Path coefficients from the longitudinal parallel mediation model.

Path/effect	b	SE	β	95% CI	*p*
Condition → adapted AEQ-derived score growth slope	0.318	0.061	0.412	[0.199, 0.437]	< 0.001
Adapted AEQ-derived score slope → Δ Self-focused attention	−0.276	0.058	−0.331	[−0.390, −0.162]	< 0.001
Adapted AEQ-derived score slope → Δ Negative self-evaluation	−0.243	0.055	−0.298	[−0.351, −0.135]	< 0.001
Δ Self-focused attention → Δ LSAS-SR	1.002	0.221	0.372	[0.569, 1.435]	< 0.001
Δ Negative self-evaluation → Δ LSAS-SR	0.789	0.193	0.336	[0.411, 1.167]	< 0.001
Mean session-level GCQ → Δ LSAS-SR	−0.112	0.18	−0.047	[−0.465, 0.241]	0.534
Condition → Δ LSAS-SR, total effect (c)	−1.569	0.712	−0.261	[−2.965, −0.173]	0.028
Condition → Δ LSAS-SR, direct effect (c′)	−1.420	0.79	−0.121	[−2.968, 0.128]	0.073
Indirect via self-focused attention	−0.088	0.026	−0.082	[−0.142, −0.041]	< 0.001
Indirect via negative self-evaluation	−0.061	0.021	−0.058	[−0.108, −0.021]	0.003
Total indirect effect	−0.149	0.037	−0.140	[−0.219, −0.086]	< 0.001

Model fit: chi-square/df = 1.34; CFI = 0.968; TLI = 0.956; RMSEA = 0.046, 90% CI [0.028, 0.063]; SRMR = 0.038.

### 4.5 Stage-dependent indirect effects

The eight sessions were divided *a priori* into adaptation (Sessions 1–2), deepening (Sessions 3–5), and integration (Sessions 6–8) stages.

Stage-specific indirect effects differed across the prespecified treatment stages (Table 6). The indirect effect was small and not statistically significant during adaptation (−0.031, 95% CI [−0.071, 0.004], *p* = 0.078), largest during deepening (−0.096, 95% CI [−0.152, −0.048], *p* < 0.001), and remained statistically significant during integration (−0.068, 95% CI [−0.118, −0.028], *p* = 0.002).

**Table 6:** Stage-specific indirect effects and pairwise contrasts.

Stage	Sessions	Indirect effect	SE	95% CI	p
Adaptation	1–2	−0.031	0.019	[−0.071, 0.004]	0.078
Deepening	3–5	−0.096	0.027	[−0.152, −0.048]	< 0.001
Integration	6–8	−0.068	0.023	[−0.118, −0.028]	0.002

Pairwise contrast	Difference	p
Deepening vs. adaptation	−0.065	0.012
Deepening vs. integration	−0.028	0.041

Negative values indicate the direction of the indirect association.

Pairwise comparisons showed a larger indirect effect during deepening than during adaptation (difference = −0.065, *p* = 0.012) and integration (difference = −0.028, *p* = 0.041). These comparisons were interpreted as exploratory tests of the prespecified treatment stages.

This ordering was consistent with H4 and suggests that the indirect association was strongest during Sessions 3–5. Because the stages were discrete and prespecified, the pattern should not be interpreted as evidence of a continuous quadratic or inverted-U function.

Figure 5 displays the stage-specific indirect effects in two complementary formats. Panel A presents signed bar estimates with confidence intervals and pairwise comparisons, whereas panel B presents the same estimates sequentially across adaptation, deepening, and integration.

**Figure 5:**
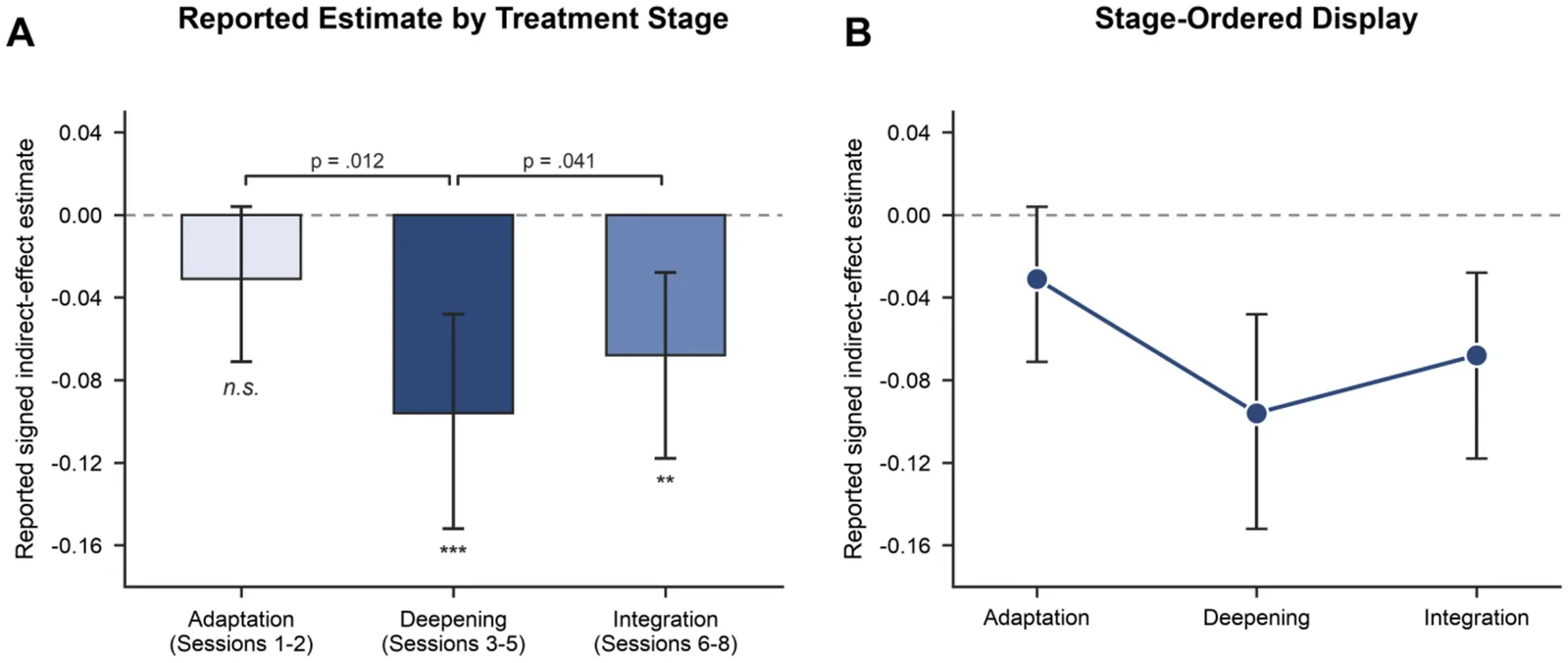
Stage-specific indirect effects across adaptation, deepening, and integration. **(A)** Signed estimates with 95% confidence intervals, within-stage significance labels, and pairwise *p*-value brackets. **(B)** The same signed estimates displayed in stage order; the connecting line is a visual guide between discrete categories and the dashed horizontal line marks zero.

The stage pattern was interpreted as exploratory evidence that the indirect association was strongest during the deepening phase, rather than as confirmation of a fixed treatment-phase mechanism.

### 4.6 Sensitivity analyses

Sensitivity analyses compared the primary intention-to-treat model with a complete-case analysis of 107 participants and an expanded multiple-imputation model using 20 imputations.

The unstandardized between-condition change estimates were directionally similar across the three analytic approaches, indicating that the estimated GAT advantage was not materially altered by the alternative missing-data specifications.

In the primary intention-to-treat analysis, the differential improvement was −12.00 points from T0 to T9 (95% CI [−16.61, −7.39], *p* < 0.001) and −13.40 points from T0 to T10 (95% CI [−18.14, −8.66], *p* < 0.001). Complete-case estimates were modestly larger in magnitude (Table 7).

**Table 7:** Sensitivity analyses for LSAS-SR treatment effects.

Analysis	*n*	Contrast	b	SE	95% CI for b	*p*
ITT with multiple imputation	120	T0 → T9	−12.00	2.35	[−16.61, −7.39]	< 0.001
ITT with multiple imputation	120	T0 → T10	−13.40	2.42	[−18.14, −8.66]	< 0.001
Complete-case analysis	107	T0 → T9	−12.74	2.41	[−17.46, −8.02]	< 0.001
Complete-case analysis	107	T0 → T10	−14.21	2.49	[−19.09, −9.33]	< 0.001
Expanded multiple imputation (m = 20)	120	T0 → T9	−11.68	2.37	[−16.33, −7.03]	< 0.001
Expanded multiple imputation (m = 20)	120	T0 → T10	−13.11	2.45	[−17.91, −8.31]	< 0.001

b is the estimated between-condition difference in change from T0; negative values favor GAT, and confidence intervals refer to b. Complete-case analysis was defined by availability of follow-up outcome data.

The expanded multiple-imputation model produced estimates similar in direction and magnitude to the primary analysis, supporting the robustness of the treatment-effect pattern across the reported missing-data approaches.

## 5 Discussion

GAT produced larger reductions in social anxiety than the supportive discussion condition, and the advantage persisted through 3-month follow-up. Session-level analyses also showed greater growth in the adapted AEQ-derived score in GAT, with exploratory indirect associations through reductions in self-focused attention and negative self-evaluation. Together, the findings extend outcome-focused art-therapy research by examining aesthetic engagement as a repeatedly measured candidate process rather than assuming it to be an established mechanism (Abbing et al., 2018, 2019; Huang et al., 2025).

The active comparator strengthens inference beyond a waitlist comparison by matching major structural and interpersonal features of treatment, including time, group size, facilitator configuration, peer contact, and opportunities for disclosure. At the same time, SDG was not inert: supportive group processes and CBT-informed prompts could themselves influence anxiety, and treatment credibility or expectancy was not measured. The programs also differed on several coupled features beyond art materials, including sensory engagement, task demands, symbolic production, and the timing and form of disclosure. The between-condition estimate therefore concerns the comparative effect of the GAT and SDG packages, not a pure effect of art-making or aesthetic experience (Mohr et al., 2009).

Clinical interpretation should remain focused on the observed group-level differences. The GAT group-mean reductions exceeded a published 28% LSAS-SR response benchmark, but cross-study comparison of aggregate means does not establish how many individual participants met responder or remission criteria. Mean endpoint scores in both conditions also remained above the published score threshold of 35; accordingly, no claim of diagnostic remission is made (von Glischinski et al., 2018). Furthermore, the primary treatment effects must be interpreted with the caveat that the analytic models did not account for facilitator or therapy-group clustering. Failure to model this non-independence may result in underestimated standard errors, potentially inflating the statistical significance of the reported treatment effects.

The candidate-process interpretation is consistent with cognitive models in which inward attention and negative self-appraisal maintain social anxiety (Rapee and Heimberg, 1997; Norton and Abbott, 2016; Adamis et al., 2025). Immersive engagement with materials may redirect attention away from self-monitoring, while artwork may permit emotionally meaningful expression at a tolerable distance. However, the study did not experimentally manipulate attention or aesthetic experience, and the adapted session score was not a direct measure of attentional allocation. The findings therefore cannot distinguish aesthetic engagement from distraction, behavioral activation, group support, expectancy, or other components of the GAT package. Crucially, because the adapted AEQ-derived measure and study-specific process measures lack adequate reliability and construct validity testing in the present sample, all process and mediation findings must be interpreted with strict caution as purely exploratory associations rather than robust mechanistic evidence. The stage ordering should be treated as a hypothesis for future replication rather than an established phase mechanism.

Repeated measurement made the temporal pattern visible but introduced burden, reactivity, and measurement-equivalence concerns (Liu et al., 2017). The original AEQ was validated for viewing art and excluded self-created art, whereas this trial used a 5-point immediate post-session adaptation in both art-making and discussion-only conditions (Wanzer et al., 2020). The meaning of the items may therefore differ by condition, and external validation studies do not by themselves establish validity for this adaptation (Świątek et al., 2023). The adapted AEQ-derived score and the study-specific self-focused-attention and negative-self-evaluation measures should therefore be interpreted as exploratory process indicators pending independent psychometric validation.

Several limitations temper the conclusions. Participants and facilitators could not be blinded, and treatment credibility or expectancy was not measured despite the active-control design. Outcomes and process variables relied heavily on self-report, and no behavioral or physiological measure directly tested the proposed attentional account. Participants were aged 18–25 years, recruited from a single Chinese university, selected on elevated self-reported symptoms without standardized diagnostic confirmation, and followed for only 3 months, limiting generalizability to clinically diagnosed samples, other age groups, and other cultural or service settings. Attrition was associated with baseline severity, and the missing-at-random assumption underlying multiple imputation remains inherently untestable. Participants were treated in therapy groups, but the analytic model did not explicitly account for therapy-group or facilitator clustering, which may affect standard-error estimation. Finally, the adapted process measures require further psychometric validation, and the follow-up period does not establish longer-term durability.

Future research should prospectively register the protocol and analysis plan, replicate the trial across institutions, cultures, age groups, and clinically diagnosed samples, and extend follow-up to at least 6–12 months. New studies should report occasion-specific reliability, test a defensible measurement model for the adapted process measures, retain therapy-group and facilitator identifiers, model clustering with appropriate small-sample corrections, assess treatment expectancy, and combine self-report with behavioral or physiological indicators. Individual LSAS-SR responder analyses, exact session descriptives, treatment-adherence estimates, and inter-rater fidelity should also be prespecified and reported. The stage-specific ordering observed here should be treated as a replication target rather than an established phase mechanism.

## 6 Conclusion

Among university students with elevated social-anxiety symptoms, eight sessions of GAT produced larger mean symptom reductions than a structurally matched supportive discussion condition, with the between-group advantage maintained at 3-month follow-up. Repeated process analyses further identified exploratory indirect associations involving growth in an adapted AEQ-derived session score, self-focused attention, and negative self-evaluation, with the strongest stage-specific association during the deepening phase. However, these process and mediation findings are severely constrained by the lack of psychometric validation for the session-level measures and should be interpreted with caution. These findings support aesthetic engagement as a plausible candidate process for further testing, but they do not establish a causal mechanism. Multi-site replication with prospectively specified analyses, independently validated process measures, and longer follow-up is needed before broader clinical or cross-cultural inference.

## Data Availability

The raw data supporting the conclusions of this article will be made available by the authors, without undue reservation.
