# Supplementary material for: Aesthetic experience as a candidate process linking group art therapy to social anxiety relief: a randomized trial with repeated process assessment
Source: Front Psychol. 2026 Sep 15;17:1953607. doi: 10.3389/fpsyg.2026.1953607 (PMC13619936; doi:10.3389/fpsyg.2026.1953607)
Supplement: Supplementary file 1 [file Table_1.DOCX]

Supplementary Material

**Supplementary Table S1：** Exact session-level descriptive statistics for the adapted AEQ-derived session score across the eight treatment sessions.

| **Session** | **Treatment Stage** | **Condition** | **N** | **Mean** | **SD** |
| --- | --- | --- | --- | --- | --- |
| **Session 1** | Adaptation | GAT | 60 | 2.21 | 0.62 |
|  |  | SDG | 60 | 2.01 | 0.58 |
| **Session 2** | Adaptation | GAT | 59 | 2.45 | 0.65 |
|  |  | SDG | 60 | 2.14 | 0.61 |
| **Session 3** | Deepening | GAT | 58 | 2.86 | 0.7 |
|  |  | SDG | 59 | 2.02 | 0.63 |
| **Session 4** | Deepening | GAT | 58 | 3.28 | 0.72 |
|  |  | SDG | 57 | 2.2 | 0.65 |
| **Session 5** | Deepening | GAT | 57 | 3.65 | 0.71 |
|  |  | SDG | 56 | 2.12 | 0.6 |
| **Session 6** | Integration | GAT | 57 | 3.89 | 0.68 |
|  |  | SDG | 54 | 2.19 | 0.62 |
| **Session 7** | Integration | GAT | 56 | 4.05 | 0.66 |
|  |  | SDG | 53 | 2.31 | 0.64 |
| **Session 8** | Integration | GAT | 55 | 4.18 | 0.65 |
|  |  | SDG | 52 | 2.19 | 0.61 |

**Supplementary CONSORT Checklist：** CONSORT 2010 checklist of information to include when reporting a randomised trial

| **Item No** | **CONSORT Item Description** | **Reported on Page/Section** |
| --- | --- | --- |
| Title and abstract |  |  |
| 1a | Identification as a randomised trial in the title | Title |
| 1b | Structured summary of trial design, methods, results, and conclusions | Abstract |
| Introduction |  |  |
| 2a | Scientific background and explanation of rationale | Section 1 & 2.1 |
| 2b | Specific objectives or hypotheses | Section 2.4 |
| Methods |  |  |
| 3a | Description of trial design (such as parallel, factorial) including allocation ratio | Section 3.1 |
| 3b | Important changes to methods after trial commencement (such as eligibility criteria), with reasons | N/A |
| 4a | Eligibility criteria for participants | Section 3.2 |
| 4b | Settings and locations where the data were collected | Section 3.2 |
| 5 | The interventions for each group with sufficient details to allow replication, including how and when they were actually administered | Section 3.5 |
| 6a | Completely defined pre-specified primary and secondary outcome measures, including how and when they were assessed | Section 3.6 & Table 1 |
| 6b | Any changes to trial outcomes after the trial commenced, with reasons | N/A |
| 7a | How sample size was determined | Section 3.3 |
| 7b | When applicable, explanation of any interim analyses and stopping guidelines | N/A |
| 8a | Method used to generate the random allocation sequence | Section 3.4 |
| 8b | Type of randomisation; details of any restriction (such as blocking and block size) | Section 3.4 |
| 9 | Mechanism used to implement the random allocation sequence (such as sequentially numbered containers), describing any steps taken to conceal the sequence until interventions were assigned | Section 3.4 |
| 10 | Who generated the random allocation sequence, who enrolled participants, and who assigned participants to interventions | Section 3.4 |
| 11a | If done, who was blinded after assignment to interventions (for example, participants, care providers, those assessing outcomes) and how | Section 3.4 |
| 11b | If relevant, description of the similarity of interventions | Section 3.5 |
| 12a | Statistical methods used to compare groups for primary and secondary outcomes | Section 3.8 |
| 12b | Methods for additional analyses, such as subgroup analyses and adjusted analyses | Section 3.8 |
| Results |  |  |
| 13a | For each group, the numbers of participants who were randomly assigned, received intended treatment, and were analysed for the primary outcome | Section 4.1 & Figure 1 |
| 13b | For each group, losses and exclusions after randomisation, together with reasons | Section 3.7 & Figure 1 |
| 14a | Dates defining the periods of recruitment and follow-up | Section 3.2 |
| 14b | Why the trial ended or was stopped | N/A (Trial completed) |
| 15 | A table showing baseline demographic and clinical characteristics for each group | Table 2 |
| 16 | For each group, number of participants (denominator) included in each analysis and whether the analysis was by original assigned groups | Section 4.3 & Table 4 |
| 17a | For each primary and secondary outcome, results for each group, and the estimated effect size and its precision (such as 95% confidence interval) | Section 4.3 & Table 4 |
| 17b | For binary outcomes, presentation of both absolute and relative effect sizes is recommended | N/A (Continuous outcomes) |
| 18 | Results of any other analyses performed, including subgroup analyses and adjusted analyses, distinguishing pre-specified from exploratory | Section 4.4, 4.5 & Table 6 |
| 19 | All important harms or unintended effects in each group | Section 3.9 (Crisis-response) |
| Discussion |  |  |
| 20 | Trial limitations, addressing sources of potential bias, imprecision, and, if relevant, multiplicity of analyses | Section 5 |
| 21 | Generalisability (external validity, applicability) of the trial findings | Section 5 |
| 22 | Interpretation consistent with results, balancing benefits and harms, and considering other relevant evidence | Section 5 & 6 |
| Other information |  |  |
| 23 | Registration number and name of trial registry | Section 5 (Acknowledged as not prospectively registered) |
| 24 | Where the full trial protocol can be accessed, if available | Section 3.9 |
| 25 | Sources of funding and other support (such as supply of drugs), role of funders | Funding Section |
